# Central Core Disease: Facial Weakness Differentiating Biallelic from Monoallelic Forms

**DOI:** 10.3390/genes13050760

**Published:** 2022-04-26

**Authors:** Ana Cotta, Lucas Santos Souza, Elmano Carvalho, Leticia Nogueira Feitosa, Antonio Cunha, Monica Machado Navarro, Jaquelin Valicek, Miriam Melo Menezes, Simone Vilela Nunes Neves, Rafael Xavier-Neto, Antonio Pedro Vargas, Reinaldo Issao Takata, Julia Filardi Paim, Mariz Vainzof

**Affiliations:** 1 The SARAH Network of Rehabilitation Hospitals, Av. Amazonas, 5953, Belo Horizonte 30510-000, MG, Brazil; ana_cotta@yahoo.com.br (A.C.); 9230@sarah.br (E.C.); 400564@sarah.br (A.C.J.); monicamnavarro@gmail.com (M.M.N.); 400585@sarah.br (J.V.); 204794@sarah.br (M.M.M.); 7713@sarah.br (S.V.N.N.); 9679@sarah.br (R.X.-N.); 204583@sarah.br (A.P.V.); 202911@sarah.br (R.I.T.); juliafilardi8@gmail.com (J.F.P.); 2 Human Genome and Stem Cells Research Center, Genetics and Evolutionary Biology, IBUSP, University of São Paulo, R. do Matao, 106, Cidade Universitária, Sao Paulo 05508-900, SP, Brazil; lucassz@usp.br (L.S.S.); leticianfp@yahoo.com.br (L.N.F.)

**Keywords:** central core disease, RYR1, clinical heterogeneity, electromyography

## Abstract

Central Core Disease (CCD) is a genetic neuromuscular disorder characterized by the presence of cores in muscle biopsy. The inheritance has been described as predominantly autosomal dominant (AD), and the disease may present as severe neonatal or mild adult forms. Here we report clinical and molecular data on a large cohort of Brazilian CCD patients, including a retrospective clinical analysis and molecular screening for *RYR1* variants using Next-Generation Sequencing (NGS). We analyzed 27 patients from 19 unrelated families: four families (11 patients) with autosomal dominant inheritance (AD), two families (3 patients) with autosomal recessive (AR), and 13 sporadic cases. Biallelic *RYR1* variants were found in six families (two AR and four sporadic cases) of the 14 molecularly analyzed families (~43%), suggesting a higher frequency of AR inheritance than expected. None of these cases presented a severe phenotype. Facial weakness was more common in biallelic than in monoallelic patients (*p* = 0.0043) and might be a marker for AR forms. NGS is highly effective for the identification of *RYR1* variants in CCD patients, allowing the discovery of a higher proportion of AR cases with biallelic mutations. These data have important implications for the genetic counseling of the families.

## 1. Introduction

Central Core Disease (CCD) is one of the most common congenital myopathies. Patients’ phenotypes range from a rarer severe neonatal myopathy similar to a congenital muscular dystrophy to a most frequent proximal weakness, manifesting in adulthood.

CCD was first described in 1956 as a congenital non-progressive myopathy characterized by hypotonia, delayed motor milestones, and a mild non-progressive weakness that predominantly affects proximal lower limb muscles [1]. This description is still very accurate, and reflects the clinical presentation of most dominantly inherited CCD patients [2].

Contrary to other congenital myopathies, CCD usually presents only discrete facial and bulbar weakness but prominent orthopedic complications such as congenital hip dislocation, Achilles tendon contractures, and joint hypermobility [3].

The histopathological hallmark of CCD is the presence of cores in the muscle fibers of the patients. Cores are areas with reduced oxidative activity observed through NADH, SDH, and COX reactions. They correspond to areas devoid of mitochondrial activity that appear on electron microscopy as round intrasarcoplasmic areas of myofibrillar disorganization with scarce or no mitochondria.

Most CCD patients present *RYR1* gene pathogenic variants. The *RYR1* gene is a huge gene with 106 exons, located at 19q13.2, with 5037 amino acids forming a 535 kDa polypeptide. This gene is also involved with the pathogenesis of other neuromuscular disorders such as Malignant Hyperthermia Susceptibility (MHS), Multiminicore disease (MmD), and Centronuclear myopathy (CNM). Malignant hyperthermia is a life-threatening condition triggered using volatile anesthetics such as halothane, and myorelaxants such as succinylcholine. Usually, CCD patients present both CCD and MHS.

The inheritance pattern of CCD is predominantly autosomal dominant, but both recessive and sporadic cases have been described [4]. The very rare severe neonatal form is characterized by the presence of profound hypotonia and has been related to *RYR1* hypomorphic variants that produce reduced levels of total RyR1 protein. These cases have been associated with recessive missense/indel *RYR1* variants [5].

The identification of *RYR1* gene variants is very challenging as more than 450 variants have been identified causing both CCD and MHS. These variants are distributed mainly in three hotspots: sarcoplasmic D1 that includes N-terminal residues 1–614, sarcoplasmic D2 that includes central region residues 2163–2458, pore-forming, SR lumen, and membrane D3 that includes the C-terminal residues 4136–4973. There is a difference between MHS and CCD predominant hotspot regions: MHS in D1 and D2, and CCD in D3 [5]. Next-generation sequencing (NGS) is a revolutionary method for *RYR1* gene study. Before NGS, the screening of *RYR1* variants was performed by Sanger Sequencing, and predominantly in the hotspot regions due to the large size of the gene and the broad distribution of the pathogenic variants. NGS has proved to be a more efficient and cost-effective technique to screen for mutations because, in addition to the *RYR1* gene, many other genes and regions can be analyzed at the same time. NGS panels may be customized according to the studied population.

The objective of this study is to describe the clinical variability in a series of Central Core Disease patients from one reference center and to correlate them with the *RYR1* gene variants and the pattern of segregation. The results have important implications for genetic counseling and for the study of physiopathological mechanisms involved in CCD.

## 2. Materials and Methods

A retrospective case series study of medical records from 1997 to 2019 has been performed, including 27 patients with Central Core Disease belonging to 19 unrelated families. Some of these patients were previously included in publications focusing on different aspects of CCD. Here, patients’ inclusion criteria were based on data from muscle biopsy with proven diagnosis of Central Core Disease and their symptomatic relatives.

### 2.1. Patients Clinical Evaluation

Medical charts review was performed with the evaluation of relevant clinical, laboratory, imaging, and muscle biopsy data.

Clinical characteristics included: age at first symptoms; age at diagnosis; gender; inheritance pattern; consanguinity; oligohydramnios; fetal akinesia; delayed motor milestones; hypotonia; bulbar weakness (sucking/swallowing); cardiac and respiratory evaluation, neonatal severe respiratory involvement; congenital hip dislocation; and club feet history.

Physical examination data included: ophthalmoplegia; palpebral ptosis; facial dysmorphisms characterized by long face and high arched palate; dolichocephaly; muscular hypotrophy; axial weakness; proximal weakness; distal weakness; and deep tendon reflexes.

Ancillary studies included electromyogram, serum total creatine kinase and aldolase levels, echocardiogram, muscle imaging, and muscle biopsy. Muscle imaging included the study of pelvis and lower limb muscles on T1-weighted axial sequences or muscle Computed Tomography in cases in which MRI could not be performed. Muscle biopsy analysis included the study of liquid nitrogen frozen specimens analyzed through the mitochondrial oxidative reactions, Succinate dehydrogenase, Cytochrome-c-oxidase, Nicotinamide adenine dinucleotide (NADH), and other routine techniques: hematoxylin and eosin, modified Gomori trichrome, Periodic acid Schiff (with and without diastase), Oil-red-O, myosinic ATPase (pH 9.4, pH 4.6, and pH 4.3), acid phosphatase, and nonspecific esterase.

### 2.2. Molecular Analysis

Patients’ DNA samples were extracted from peripheral blood lymphocytes using a routine methodology.

The genetic investigation was performed firstly by using an NGS customized panel for 95 neuromuscular diseases (NMD) genes including the *RYR1* gene. Afterwards, we used the llumina TruSight One Expanded panel, which targets more than 6700 genes and exonic regions associated with clinical phenotypes.

The following preparation kits used were: SureSelect OXT library and SureSelect Human all exons and V6 capture kit (Agilent, Santa Clara, CA, USA). The sequencing was performed on Hiseq2500 equipment (Illumina, San Diego, CA, USA). The human genome reference for data alignment was the version GRCh37/hg19.

A control population of 1000 Genomes, NIH, gnomAD, 6500 Exaomes Sequencing Project (Washington University, Washington, DC, USA), and the new AbraOM (Online Archive of Brazilian Mutations) were used for comparison with the filtered variants. The *RYR1* gene (OMIM#180901, transcript NM_000540.3) rare variants were checked and analyzed using bioinformatic tools. Gene Mutations Databases HGMD, LOVD, and ClinVar were checked for already described pathogenic *RYR1* variants. The American College of Medical Genetics and Genomics (ACMG) pathogenicity classification guidelines [6] were used for variants classifications. The prediction of pathogenesis of de novo variants was supported evaluating the results from many in silico prediction softwares: MutationTaster, Predict SNP1, CADD, DANN, FATHMM, FunSeq2, GWAVA, VEP, SIFT, Polyphen2, and Human splicing finder 3.0.

For the confirmation of pathogenic variants, relatives screening, variants segregation study within the families, and Sanger Sequencing of specific exons were performed.

### 2.3. Data Analysis

Data analysis was performed with approval of the institutional Scientific and Ethical Committees. Statistical descriptive analysis and comparison between clinical groups were performed using the Fisher exact test with statistical significance for *p* < 0.05.

## 3. Results

**Figure 1:**
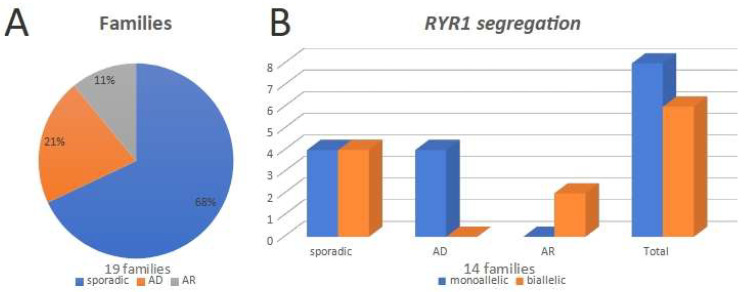
(**A**)—Distribution of the studied families, according to the pedigrees. (**B**)—Distribution of the 14 families with genetic studies, according to the presence of mono or biallelic variants in *RYR1*.

A total of 27 patients from 19 families were selected. Sporadic cases were the most common, being half of the cases (13/27 patients), followed by autosomal dominant (11 patients from four families) and recessive inheritances (three patients from two families) (Figure 1A).

**Figure 2:**
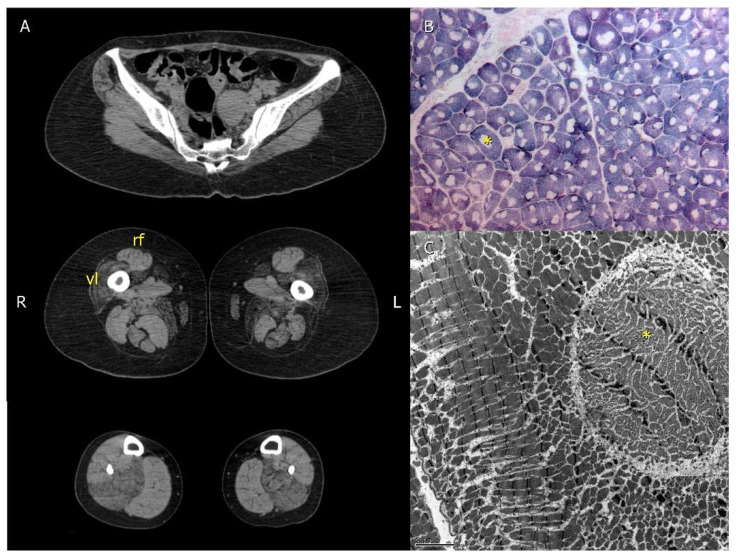
Imaging and muscle biopsy findings of patient 2.1 with the p.Gly4897Asp pathogenic variant in the *RYR1* gene. (**A**)—Computed tomography demonstrated severe right *vastus lateralis* (vl) muscle fat replacement with relative *rectus femoris* (rf) preservation. Computed tomography of the pelvis, thighs, and legs. (**B**)—Muscle biopsy was performed in the *rectus femoris* demonstrating round core structures (*). SDH 100x. (**C**)—Core areas (*) were ultrastructurally characterized by myofibrillar disorganization with scarce mitochondria. Transmission electron microscopy 2500×. This figure was modified from Cotta et al., 2021 [6].

Imaging studies were used to guide the choice for the best site for muscle biopsy. Due to the frequent muscle fat replacement of the *vastus lateralis*, the *rectus femoris* was the muscle of choice whenever the former was involved (Figure 2A).

All patients submitted to muscle biopsy presented areas devoid of oxidative reaction correspondent to myofibrillar disorganization with scarce mitochondria (Figure 2B,C).

A summary of clinical, laboratory, and molecular data is provided in Table 1, Table 2 and Table 3. Sixteen patients were female and eleven were male. Most patient presented hypotonia (14/23) and developmental delay (15/23) (Table 1).

The mean age at diagnosis was 18 ± 15.8 years and the age range varied from 1 to 52 years. Only one patient reported first symptoms in adulthood (Table 1). All other patients presented first symptoms in childhood.

Congenital hip dislocation was observed in 5/25 patients and congenital club feet in 4/26 patients. Bulbar symptoms, characterized by difficult sucking or swallowing, were observed in 3/15 patients and severe neonatal respiratory involvement was reported in 2/17 patients.

No signs of significant cardiac or pulmonary abnormalities were observed: all patients presented normal cardiac and pulmonary auscultation. Nevertheless, all of them received recommendation to be submitted to cardiac evaluation at external specialized services.

Facial weakness was observed in 8/27 patients and facial dysmorphisms, characterized by long face, high arched palate, and dolichocephaly were observed in 6/27 patients. Palpebral ptosis was observed in 2/28 patients and none of them presented ophthalmoplegia.

The distribution of muscle weakness was predominantly proximal in 96% (25/26) of the patients, while distal (26%, 7/26) and axial (32%, 8/25) weakness were least frequent.

Deep tendon reflexes were absent (45%, 11/24) or decreased (29%, 7/24) in most patients. Five patients (21%, 5/24) presented normal reflexes. One isolated patient (Patient 19.1 in Table 2) presented increased reflexes. Electromyogram was able to detect either a myopathic pattern or the suggestion of myopathic motor unit potentials in 88% (21/24) of the patients. Only two patients (8%, 2/24) presented normal electromyogram. One patient (Patient 11.1) presented mixed myopathic and neurogenic motor unit potentials (Table 2).

Serum total creatine kinase levels were normal or almost normal in 84% (22/26) of the patients. One patient (18.1 in Table 2) presented 4.3 times (966 IU/L) increased total creatine kinase levels (Table 2).

A total of 21 variants in the *RYR1* gene were identified in the 14 molecularly studied families (five sporadic cases were not available for the molecular analysis). Two variants were recurrent: p.Ala4846Val in two families (#5, #6) and p.Arg4861His in three families (#8, #10, #13) (Table 3).

**Figure 3:**
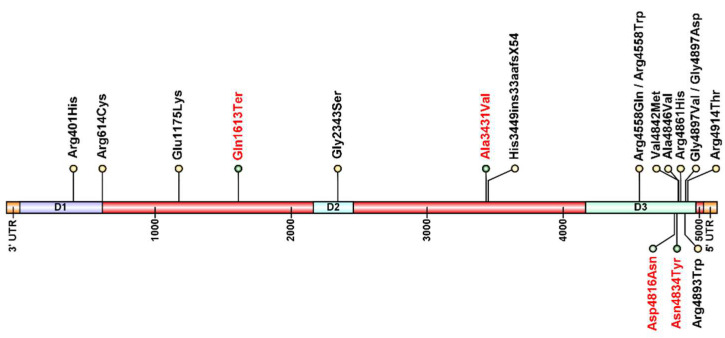
Diagram of the variants in the *RYR1* gene in this cohort of patients. Novel variants identified in this work are depicted in red and previously described variants are represented in black. D1, D2, and D3 are the three hotspots for mutations in the *RYR1* gene, and the majority of the variants identified in this cohort are located at D3. Figure created using IBS Illustrator for Biological Sequences [19].

Among the 18 different mutations, 16 were missense nonsynonymous variants, one was a nonsense variant, and one was an intronic variant. Fourteen of the variants were previously described as pathogenic [5,7,8,9,10,11,12,13,14,15,16,17,18] and four variants are now being associated with the CCD phenotype. Eleven of the mutations were localized in the C-region, while three were in hotspot D1 and D2 N-terminal and sarcoplasmic domains of the RyR1 channel, and four variants were outside of any of the three main domains (Figure 3).

Molecular analysis disclosed monoallelic variants in the four AD families and in four sporadic cases, while biallelic variants were found in the two AR families (three patients) and in four sporadic cases (Figure 1B). Therefore, genotypic analysis disclosed 8/14 (57%) families with monoallelic variants (15 patients: 11 AD and four sporadic) and 6/14 (43%) families with biallelic variants (seven patients: three AR and four sporadic). Only one family was consanguineous; however, all the biallelic mutations were compound heterozygous (Table 3).

**Figure 4:**
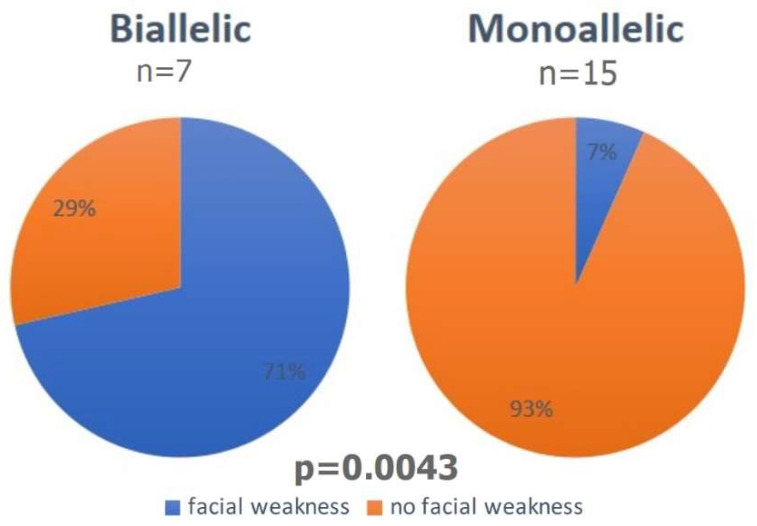
Facial weakness in biallelic and monoallelic *RYR1* families.

A comparison between the phenotypes found in monoallelic versus biallelic patients was performed. There were no statistically significant differences between both groups considering the frequency of facial dysmorphism, axial weakness, distal weakness, hypotrophy, bulbar symptoms, congenital club feet, severe neonatal respiratory distress, or hip dislocation (Table 4). However, facial weakness was significantly more frequent in the biallelic (71.0% or 5/7) than the monoallelic (6.7% or 1/15) patients (*p* = 0.0043) (Figure 4) (Table 4).

## 4. Discussion

Central Core Disease is the most common cause of congenital myopathy, morphologically defined by cores on muscle biopsy, which can be unique or multiple, peripheral or central, clear areas on oxidative reactions that extend the longitudinal length of the myofiber [20,21,22,23,24]. All patients included in this study (or at least one patient from each family) showed the presence of cores in the muscle biopsy, confirming the diagnosis of CCD.

All the patients, except for one (Table 1), reported first symptoms in childhood. Most patients whose diagnosis was performed in adulthood reported decreased physical performance compared to other children. Orthopedic abnormalities have been previously described in ryanodinopathy [5,7,13].

In this series, congenital hip dislocation was observed in 20% of the patients (5/25) and club feet in 15% (4/26) when this data was available. Orthopedic abnormalities are a common feature in CCD, thus our data are in accordance with the literature [3,20]. Genetic evaluation of the patients with congenital hip dislocation and congenital club feet may suggest underlying congenital myopathy.

Pes cavus was identified in two patients (11.5%, 3/26). This is an unexpected finding as this physical characteristic is commonly reported in nemaline congenital myopathy and centronuclear myopathy [20].

Palpebral ptosis was observed in 2/27 and neither of them presented ophthalmoplegia. This finding is in accordance with previous publications, mainly because ophthalmoplegia is more common in patients with centronuclear and multiminicore histological diagnoses and not CCD [20].

Serum creatine kinase levels were either within or close to the normal range in all but one patient (Patient 18.1). This patient had a history of previous episodes of malignant hyperthermia.

An electromyogram successfully determined the myopathic nature of the disease process in 88% (21/24) of the patients. This is in accordance with a previous study [25] that reported when an electromyogram is performed by experienced professionals, with special skills in neuromuscular disorders, it provides invaluable help with diagnosis.

Based on the data available in the medical charts, information about an echocardiogram was registered in 5/26 patients. The echocardiogram was completely normal in 4/5 patients. Patient 3.1 with the p.Asn4834Tyr pathogenic variant presented preserved systolic/diastolic biventricular function with slight mitral regurgitation with mitral prolapse.

Information about an electrocardiogram was registered in 15/26 patients. The result was completely normal in 13/15 patients. Patient 2.1 with the p.Gly4897Asp pathogenic variant presented a slight left deviation in electrocardiogram. Patient 5.2 with two pathogenic variants (p.Arg4558Gln and p.Ala4846Val) presented slow right bunch conduction.

No patient presented any signs of respiratory involvement as a characteristic clinical finding of Central Core Disease [26].

Pathogenic variants in the ryanodine receptor (*RYR1*) gene are the most common causes of congenital myopathy, and the most frequent cause of Central Core Disease [21,27]. With the introduction of NGS testing and the possibility to screen all the 108 exons of the *RYR1* gene, the capacity to detect mutations was significantly improved. In fact, although a predominance of variants in the C-terminal D3 domain (11/18 variants) is still observed, three variants were localized in domains D1 and D2 and an additional four variants (two novel) were found outside of these three hotspot domains. These findings strengthen the knowledge that CCD mutations are indeed located in the D3 domains, but also point to the presence of pathogenic variants outside these regions, showing the importance of sequencing the whole *RYR1* gene to identify such variants that would have been missed if only the hotspots regions were sequenced.

Genetically, most of our CCD patients present autosomal dominant mild, non-progressive limb weakness, as already described [21]. Nevertheless, although severe CCD with respiratory and bulbar symptoms was usually described in recessive cases [21], in this series, only 3/18 patients presented severe bulbar symptoms, and their inheritance pattern was either autosomal dominant or sporadic. Two severe cases (16.1 and 19.1) were not submitted to molecular analysis.

Additionally, none of the patients from our six families with biallelic variants presented a severe phenotype with bulbar weakness. These results suggest that with the augmentation of the capacity of the molecular identification of CCD cases with biallelic mutations, the clinical phenotype is exhibiting a broader variability, with milder cases described recently.

As to the genotype–phenotype correlations, there were some clinical and morphological differences between the patients here described and previous publications of the same *RYR1* variants. The p.Arg401His and p.Arg614Cys variants were previously described as monoallelic, causing disease mutations in patients with malignant hyperthermia [16,17]. We present here a case of a patient (12.1) in which the combination of these two variants causes a Central Core Disease phenotype, being an example of phenotypic variability associated with *RYR1* mutations.

The p.Ala4846Val (exon 101) variant was previously described both in Central Core Disease and in an autosomal recessive centronuclear *RYR1*-related congenital myopathy [7,11]. Both conditions presented with developmental delay and hypotonia. However, facial weakness without ophthalmoplegia was described in Central Core Disease [7], while ophthalmoplegia without facial weakness was reported in *RYR1*-related centronuclear myopathy [11]. The variant p.Gly4897Asp was previously described both as a monoallelic and biallelic disease [5,28], meaning that this variant can cause a dominant phenotype when present in heterozygosis, probably associated with a second genetic modification that predisposes to the disease, but also a recessive phenotype when in homozygosis. Therefore, clinical variability can be detected in patients carrying the same mutations, suggesting the action of other factors modulating the phenotype.

Regarding the pathogenicity of the novel mutations here identified, three out of four were classified as likely pathogenic (if we assume that they are de novo variants but without confirmation of paternal and maternal segregation): the variant p.Asn4834Ty (PM2_Strong, PM6, PP3), the variant p.Asp4816Asn (PM2_Strong, PM5, PM6, PP3), and the variant p.Ala3431Val (PM2_Strong, PM6, PP3). The variant p.Ala3431Val was found only in Patient 13.1, who also has the pathogenic p.Arg4861His described mutation found in heterozygosis in another two of our sporadic patients (8.1 and 10.1), as well as in another report [13]. This suggests that, although the novel variant p.Ala3431Val might be pathogenic, the main mutation causing CCD in the patient is p.Arg4861His. The remaining fourth novel mutation p.Gln1613Ter causes a truncated protein so, as expected, it received a pathogenic classification (PVS1, PM2, PP3, PP5). This variant was found in association with a second previously described mutation p.Glu1175Lys [15], which was classified as a VUS (Variant of Uncertain Significance) by the authors. Once the p.Gln1613Ter mutation is sporadic, we cannot conclude whether it alone is sufficient to cause a CCD phenotype in a dominant manner, or if the presence of another mutation is mandatory.

Interestingly, Patient 6.1 has the same two mutations (p.Gly2343Ser and p.Arg4558Trp) that were identified in three different patients [10,11] also in compound heterozygosis with an autosomal recessive inheritance. As with our patient, both patients reported by Samões, 2017 also had a third mutation at *RYR1* [10].

A comprehensive multiprofessional orthopedic and genetic evaluation of these patients may provide valuable clinical orientation and genetic counseling..

Comparing mono versus biallelic patients, only facial weakness was able to differentiate both groups with statistical significance. This important clinical signal can thus direct the analysis of new cases, aiming at genetic counseling.

Finally, in this large cohort of patients from the same reference center, it was not possible to infer that biallelic cases presented more severe phenotypes than monoallelic cases. On the other hand, it provided some evidence that facial weakness might be a clue to biallelic inheritance.

## 5. Conclusions

NGS is improving our capacity to identify mutations in the *RYR1* gene and increasing the number of identified pathogenic mutations, either as a heterozygous allele or compound heterozygous alleles. This is the probable reason for the increasing number of biallelic cases than previously reported. Facial weakness was more common in biallelic than in monoallelic patients in this group, and it could be a marker for AR forms. These data have important implications for the genetic counseling of the families.

## Figures and Tables

**Table 1:** Clinical characteristics of 27 Central Core Disease patients.

Fam	Patient	Inherit.	Start	Age	Gender	Oligo.	Akinesia	Delay	Hypotonia	Bulbar	Resp.	Hip Disl.	Club Feet
1	1.1	D	0	37	M	NR	NR	Y	Y	N	N	N	N
1	1.2	D	28	28	F	N	N	Y	Y	N	N	N	N
1	1.3	D	0	8	M	N	N	Y	Y	N	N	N	N
1	1.4	D	0	1	M	N	N	Y	Y	N	N	N	N
2	2.1	D	0	24	F	NA	NA	Y	NA	NA	NA	NA	NA
2	2.2	D	0	5	M	N	N	Y	Y	Y	Y	N	N
3	3.1	D	1	25	F	NA	NA	N	N	NA	NA	N	N
3	3.2	D	NR	47	F	NA	NA	NA	NA	NA	NA	NA	NA
4	4.1	D	2	18	F	NR	NR	N	NR	NR	NR	N	N
4	4.2	D	2	26	M	NR	NR	NR	NR	NR	NR	N	N
4	4.3	D	2	52	F	NR	NR	NR	NR	NR	NR	N	N
5	5.1	R	8	38	M	N	N	N	N	N	N	N	N
5	5.2	R	7	42	F	N	N	N	N	N	N	N	Y
6	6.1	R	0	1	F	N	N	Y	Y	N	N	Y	N
7	7.1	S	2	27	M	NR	NR	N	N	N	N	N	N
8	8.1	S	0	7	F	Y	N	Y	Y	N	N	Y	Y
9	9.1	S	0	17	M	N	N	N	Y	N	N	N	N
10	10.1	S	2	14	M	NR	NR	NR	NR	NR	NR	NR	Y
11	11.1	S	0	7	M	NR	NR	N	N	N	N	N	N
12	12.1	S	0	2	F	Y	N	Y	Y	N	N	Y	N
13	13.1	S	0	4	M	N	N	Y	Y	N	N	Y	N
14	14.1	S	0	9	F	N	N	Y	Y	N	N	N	N
15	15.1	S	0	4	F	N	Y	Y	Y	N	N	Y	N
16	16.1	S	0	5	F	NR	NR	Y	Y	Y	NR	N	N
17	17.1	S	0	41	F	NR	NR	Y	NR	NR	NR	N	N
18	18.1	S	0	9	F	N	N	N	N	N	N	N	Y
19	19.1	S	0	2	F	N	N	Y	Y	Y	Y	N	N

Fam: family number. P: Patient. Start: age (years) of first symptoms. Age: age (years) at diagnosis. Gender: female (F), male (M). Inherit.: Inheritance: autosomal dominant (D), autosomal recessive (R), sporadic (S). Cons.: consanguineous. Oligo.: Oligohydramnios: yes (Y), no (N). Akinesia: fetal akinesia: yes (Y), no (N). Delay: delayed motor milestones: yes (Y), no (N). Hypotonia: yes (Y), no (N). Bulbar: Bulbar weakness (sucking/swallowing): yes (Y), no (N). Resp.: Neonatal severe respiratory involvement: yes (Y), no (N). Hip Disl.: hip dislocation: yes (Y), no (N). Club Feet: yes (Y), no (N). NR: not reported. NA: not available.

**Table 2:** Physical, neurophysiological, laboratorial findings in 27 Central Core Disease patients.

F	P	I	Face	Oph	Pt	Dys	Hypo	Ax	Prox	Dist	Reflex	ENMG	CK (×)	Other Findings
1	1.1	D	N	N	N	N	Y	N	Y	N	A	M	63 (<1×)	
1	1.2	D	N	N	N	N	N	N	Y	N	NR	M	75 (<1×)	
1	1.3	D	N	N	N	N	Y	N	Y	N	A	M	75 (<1×)	
1	1.4	D	N	N	N	N	N	Y	Y	N	A	NA	102 (<1×)	
2	2.1	D	N	N	N	N	Y	N	Y	N	A	M	267 (1.6×)	
2	2.2	D	N	N	N	N	Y	Y	Y	Y	A	M	131 (<1×)	Knee contractures
3	3.1	D	N	N	N	N	Y	N	Y	N	NL	M	74 (<1×)	Ankle contracture
3	3.2	D	N	N	N	N	N	NA	NA	NA	NA	NA	NA	
4	4.1	D	N	N	N	N	N	N	Y	N	NL	M	156 (<1×)	Right calf atrophy, winged scapula
4	4.2	D	N	N	N	N	N	N	Y	N	NA	M	62 (<1×)	
4	4.3	D	N	N	N	N	N	N	Y	N	NL	NA	78 (<1×)	
5	5.1	R	Y	N	N	N	N	N	Y	N	Hypo	M	189 (1×)	
5	5.2	R	N	N	N	N	N	N	Y	N	Hypo	M	389 (2.8×)	Toe walking
6	6.1	R	Y	N	N	Y	N	Y	Y	N	Hypo	M	52 (<1×)	
7	7.1	S	N	N	N	N	Y	N	Y	N	Hypo	M	51 (<1×)	
8	8.1	S	Y	N	N	N	Y	N	Y	Y	A	M	93 (<1×)	Gait with orthesis at age 5
9	9.1	S	N	N	N	N	Y	N	Y	N	A	M	50 (<1×)	No gait acquisition
10	10.1	S	N	N	N	N	N	Y	Y	N	Hypo	M	39 (<1×)	Pes cavus
11	11.1	S	Y	N	N	N	N	Y	Y	Y	A	Mix	79 (<1×)	
12	12.1	S	N	N	N	N	N	Y	Y	Y	A	M	65 (<1×)	Pes cavus, knee contractures
13	13.1	S	Y	N	N	Y	Y	N	Y	N	Hypo	M	36 (<1×)	Joint laxity
14	14.1	S	Y	N	Y	Y	Y	NA	Y	N	A	M	33 (<1×)	Hydrocephalia, joint laxity
15	15.1	S	N	N	N	N	Y	N	Y	N	Hypo	NL	49 (<1×)	Scoliosis, Left knee contracture
16	16.1	S	Y	N	Y	Y	Y	Y	Y	Y	A	M	80 (<1×)	
17	17.1	S	N	N	N	N	N	N	Y	Y	NL	M	293 (1.7×)	Pes cavus. Knee dislocation
18	18.1	S	N	N	N	Y	N	N	Y	Y	NL	M	966 (4.3×)	Malignant hyperthermia episode. Small mouth
19	19.1	S	Y	N	N	Y	N	Y	Y	N	Hyper	NL	67 (<1×)	Café-au-lait spots

F: family. P: patient. I: Inheritance. Face: Facial weakness: yes (Y), no (N). Oph: ophthalmoplegia: yes (Y), no (N). Pt: palpebral ptosis: yes (Y), no (N). Dys: facial dysmorphisms characterized by long face, high arched palate, dolichocephaly: yes (Y), no (N). Hypo: muscular hypotrophy: yes (Y), no (N). Ax: axial weakness: yes (Y), no (N). Prox: proximal weakness: yes (Y), no (N). Dist: distal weakness: yes (Y), no (N). Reflex: deep tendon reflexes: normal (N), absent (A), decreased/hypoactive (Hypo), increased/hyperactive (Hyper). ENMG: electromyogram: myopathic (M), neurogenic (N), mixed myopathic and neurogenic (Mix), normal (NL), not performed/not available (NA). CK (x): serum total creatine levels (times increase). NA: not available or not performed.

**Table 3:** *RYR1* variants in Central Core Disease families.

Family	Patient	Inheritance	Variant	Protein Change	Exon	Reference
1	1.1	Dominant	c.14690G>T	p.Gly4897Val	102	Kossugue et al., 2007 [7]. Galleni-Leao et al., 2020 [8]
2	2.1	Dominant	c.14690G>A	p.Gly4897Asp	102	Amburguey et al., 2013 [5]
3	3.1	Dominant	**c.14500A>T**	**p.Asn4834Tyr**	100	**Novel**
4	4.3	Dominant	**c.14446G>A**	**p.Asp4816Asn**	100	**Novel**
5	5.2	Recessive	c.13673G>A	p.Arg4558Gln	94	Kossugue et al., 2007 [7]
			c.14537C>T	p.Ala4846Val	101	Galleni-Leao et al., 2020 [8]. Gambelli et al., 2007 [9]
6	6.1	Recessive	c.7027G>A	p.Gly2343Ser	43	Samões et al., 2017 [10]. Abath-Neto et al., 2017 [11]
			c.13672C>T	p.Arg4558Trp	94	Samões et al., 2017 [10]. Abath-Neto et al., 2017 [11]
			c.14537C>T	p.Ala4846Val	101	Galleni-Leao et al., 2020 [8]. Gambelli et al., 2007 [9]
7	7.1	Sporadic	c.14677C>T	p.Arg4893Trp	101	Cotta et al., 2017 [12]. Galleni-Leão et al., 2020 [8]
8	8.1	Sporadic	c.14582G>A	p.Arg4861His	101	Monnier et al., 2001 [13]
9	9.1	Sporadic	c.14741G>C	p.Arg4914Thr	102	Galleni-Leão et al., 2020 [8]. Davis et al., 2003 [14]
10	10.1	Sporadic	c.14582G>A	p.Arg4861His	101	Galleni-Leão et al., 2020 [8]. Monnier et al., 2001 [13]
11	11.1	Sporadic	c.3523G>A	p.Glu1175Lys	26	Chae et al., 2015 [15]
			**c.4837C>T**	**p.Gln1613Ter**	33	**Novel**
12	12.1	Sporadic	c.1202G>A	p.Arg401His	12	Rueffert et al., 2002 [16]
			c.1840 C>T	p.Arg614Cys	17	Gillard et al., 1991 [17]
13	13.1	Sporadic	**c.14292C>T**	**p.Ala3431Val**	68	**Novel**
			c.14582G>A	p.Arg4861His	101	Monnier et al., 2001 [13]
14	14.1	Sporadic	c.10348-6C>G	p.His3449ins33aafsX54	-	Monnier et al., 2008 [18]
			c.14524G>A	p.Val4842Met	101	Monnier et al., 2008 [18]
15	15.1	Sporadic	-	-	-	-
16	16.1	Sporadic	-	-	-	-
17	17.1	Sporadic	-	-	-	-
18	18.1	Sporadic	-	-	-	-
19	19.1	Sporadic	-	-	-	-

Family: family number. Patient: Patient number. Bold: *RYR1* gene variants now associated with CCD. Underlined variants: recurrent variants in different families.

**Table 4:** Clinical characteristics in monoallelic and biallelic *RYR1* variants.

	Monoallelic			Biallelic			
Clinical Characteristic	Percentage	Yes	*n*	Percentage	Yes	*n*	*p* Value
**Facial weakness**	**6.7%**	**1**	**15**	**71.0%**	**5**	**7**	***p* = 0.0043**
Facial dysmorphism	0%	0	15	42.9%	3	7	*p* = 0.0227
Axial weakness	21.4%	3	14	50.0%	3	6	*p* = 0.3027
Distal weakness	14.3%	2	14	28.6%	2	7	*p* = 0.5743
Hypotrophy	53.3%	8	15	28.6%	2	7	*p* = 0.3808
Bulbar symptoms	12.5%	1	8	0%	0	7	*p* = 1.0000
Congenital club feet	15.4%	2	13	14.3%	1	7	*p* = 1.0000
Neonatal respiratory	12.5%	1	8	0%	0	7	*p* = 1.0000
Hip dislocation	8.3%	1	12	42.9%	3	7	*p* = 0.1174

Bold: statistically significant difference between monoallelic and biallelic patients.

## Data Availability

All data generated or analyzed during this study are included in this published article.
